# Data on preschool children׳s math, patterning, and spatial knowledge

**DOI:** 10.1016/j.dib.2018.07.061

**Published:** 2018-07-31

**Authors:** Bethany Rittle-Johnson, Erica L. Zippert, Katherine L. Boice

**Affiliations:** Vanderbilt University, United States

## Abstract

Initial participants were 79 children who were recruited from six preschool programs in the U.S. Full assessment data was available for 73 children (average age of 4 years 7 months), including demographic data (gender, ethnicity, financial need, language(s) spoken at home and special education status). Children׳s math, repeating patterning, spatial and verbal skills were assessed at the beginning of the pre-kindergarten year. Assessments included the brief version of the *Research-Based Early Mathematics Assessment,* two measures of repeating patterning skills, three measures of spatial skills (the Block Design subtest of the *Wechsler Preschool and Primary Scale of Intelligence*, the Position in Space subtest of the *Developmental Test of Visual Perceptio*n, and a Corsi Block Tapping Task), the Picture Vocabulary Test from the *NIH Toolbox app* and a backward letter span task. Near the end of the school year, their math knowledge was re-assessed using the same math measure, as was their memory span (forward and backward digit span task from the *Wechsler Intelligence Scale for Children).* Findings on the relations between patterning, spatial and math skills are published elsewhere (Rittle-Johnson et al., 2018) [1].

**Specifications Table**

**Table:** 

Subject area	*Psychology, Education*
More specific subject area	*Cognitive Development*
Type of data	*text files of data*
How data was acquired	*Individual assessment of children by research assistants, as used in Rittle-Johnson et al.**[1]*
Data format	*Raw data files of item-level data and of summary scores*
Experimental factors	*For some measures, Item Response Analysis was used to generate ability estimates for children.*
Experimental features	*Data was acquired through individual assessment of preschool children׳s math, repeating patterning, spatial and verbal skills*
Data source location	*Nashville, TN, USA*
Data accessibility	*Data are with this article*
Related research article	B. Rittle-Johnson, E. Zippert, K. Boice, The roles of patterning and spatial skills in early mathematics development. *Early Childhood Research Quarterly*, (in press) 10.1016/j.ecresq.2018.03.006.

**Value of the data**

• Item-by-item level data can be used to better understand children׳s knowledge and to refine measures of preschool children׳s knowledge and skills, especially for math and repeating patterns.
• Data can be used to investigate relations between different types of knowledge and skills in early childhood.
• Data can be used to investigate whether children with different demographic characteristics vary in their early knowledge and skills.

## 1 Data

The PatternSpatial_Measures.txt data file includes children׳s overall score on each of the measures that were administered as well as available demographic characteristics of the children. The PatternSpatial_ItemLevelData.txt file includes item-by-item level data on a subset of measures were such data is available (research pattern, teacher pattern, REMA Time 1, REMA Time 2).

## 2 Experimental design, materials and methods

The study used a longitudinal design. This data was used in Rittle-Johnson et al. [1], and details about the participants, procedure and measures are provided in that publication.

### 2.1 Participants

Data is from 73 children, with an average age of 4 years 7 months (*SD* = 4 months; range = 4 years 0 months – 5 years 2 months) when first assessed. The sample was ethnically and economically diverse, with additional information available in [1].

### 2.2 Procedure

All procedures were reviewed and approved by the Vanderbilt University Institutional Review Board Committee. Within the first quarter of the school year, children were individually assessed at their preschools in two sessions using all measures described below except the digit span task. During the final quarter of the school year, children were assessed in a single session on the math and digit span tasks.

### 2.3 Measures

#### 2.3.1 Repeating patterning skills

##### 2.3.1.1 Research-based patterning assessment

This assessment consisted of nine items at four levels of difficulty designed to measure preschoolers’ ability to duplicate, extend, abstract, and identify units of repeating visual patterns. Ability estimates for children were generated using a Rasch model with a Laplace approximation and empirical Bayesian prediction method that has been shown to be stable for sample sizes around 50 [2], implemented in R (http://www.r-project.org), using the glmer function of the lme4 package [3]. The R code is included with the Data in Brief in the Pattern Measure IRT R Code file.

##### 2.3.1.2 Teacher-based patterning assessment

This 10-item assessment was developed using pre-existing patterning worksheets found on websites with resources for early-childhood educators with four types of tasks: what comes next, missing item, extending patterns and matching patterns. Ability estimates were generated using a Rasch model with a Laplace approximation method. The R code is included with the Data in Brief.

### 2.3.2 Spatial skills

Three established measures of spatial skills were used: (a) The Position in Space subtest of the *Developmental Test of Visual Perceptio*n–*Second Edition* [4], (b) Block Design, a subtest of the *Wechsler Preschool and Primary Scale of Intelligence*–*Fourth Edition* [5] and (c) the Corsi Block Tapping Task, implemented using the *PathSpan* program on an iPad (available at https://hume.ca/ix/pathspan.html). Scores on the Corsi task were the number of trials correct on both the forward and backward order of the task.

One child (subject 103) scored over 4 standard deviations above the mean on the Corsi block tapping task, and 2 standard deviations above the next highest score. This child often scored 2 standard deviations above the mean on other measures. This student is flagged as “recommended to be dropped from analyses” in the data files.

### 2.3.3 Math knowledge

The REMA Short-Form contains 19 items, split into two sections: 13 items assessing children׳s numeracy knowledge, and 6 items assessing their geometric knowledge [6]. IRT ability estimates were generated using a partial credit model.

### 2.3.4 Verbal ability

The Picture Vocabulary Test from version 1.6 of the *NIH Toolbox app* assessed children׳s receptive vocabulary and age-corrected and standardized scores are provided.

### 2.3.5 Verbal short-term and working memory

At Time 1, a backward letter span task was administered [7] based on the backwards digit span task from the *Wechsler Intelligence Scale for Children*, but 60% of children failed to complete a single trial correctly. The forward and backward digit span task from the *Wechsler Intelligence Scale for Children* was administered at Time 2 [8]. Separate scores for the forward and backward orders are provided, calculated by summing the number of total trials answered correctly for each order.
